# Removal of the blue component of light significantly decreases retinal damage after high intensity exposure

**DOI:** 10.1371/journal.pone.0194218

**Published:** 2018-03-15

**Authors:** Javier Vicente-Tejedor, Miguel Marchena, Laura Ramírez, Diego García-Ayuso, Violeta Gómez-Vicente, Celia Sánchez-Ramos, Pedro de la Villa, Francisco Germain

**Affiliations:** 1 Departamento de Biología de Sistemas, Facultad de Medicina, Universidad de Alcalá, Alcalá de Henares, Madrid, Spain; 2 Departamento de Óptica, Facultad de Óptica, Universidad Complutense, Madrid, Spain; 3 Departamento de Oftalmología, Facultad de Medicina, Universidad de Murcia, Murcia, Spain; 4 Instituto Murciano de Investigación Biosanitaria- Hospital Virgen de la Arrixaca (IMIB-Arrixaca), Murcia, Spain; 5 Departamento de Óptica, Farmacología y Anatomía, Universidad de Alicante, Alicante, Spain; Dalhousie University, CANADA

## Abstract

Light causes damage to the retina (phototoxicity) and decreases photoreceptor responses to light. The most harmful component of visible light is the blue wavelength (400–500 nm). Different filters have been tested, but so far all of them allow passing a lot of this wavelength (70%). The aim of this work has been to prove that a filter that removes 94% of the blue component may protect the function and morphology of the retina significantly. Three experimental groups were designed. The first group was unexposed to light, the second one was exposed and the third one was exposed and protected by a blue-blocking filter. Light damage was induced in young albino mice (p30) by exposing them to white light of high intensity (5,000 lux) continuously for 7 days. Short wavelength light filters were used for light protection. The blue component was removed (94%) from the light source by our filter. Electroretinographical recordings were performed before and after light damage. Changes in retinal structure were studied using immunohistochemistry, and TUNEL labeling. Also, cells in the outer nuclear layer were counted and compared among the three different groups. Functional visual responses were significantly more conserved in protected animals (with the blue-blocking filter) than in unprotected animals. Also, retinal structure was better kept and photoreceptor survival was greater in protected animals, these differences were significant in central areas of the retina. Still, functional and morphological responses were significantly lower in protected than in unexposed groups. In conclusion, this blue-blocking filter decreases significantly photoreceptor damage after exposure to high intensity light. Actually, our eyes are exposed for a very long time to high levels of blue light (screens, artificial light LED, neons…). The potential damage caused by blue light can be palliated.

## Introduction

Light is converted into useful visual information in the retina. Photoreceptor cells express light-sensitive pigments that absorb photons, initiating a chemical cascade of events known as phototransduction that culminates in the generation of electrical signals. There are three classes of retinal cells that contain visual pigments and are thus responsive to light: the classic photoreceptors, rods and cones, and the intrinsically-photosentitive retinal ganglion cells (ipRGCs). Rods and cones contain rhodopsin and cone opsins respectively, allowing visual perception and color distinction, whereas ipRGCs contain melanopsin and are involved in the entrainment of the circadian rhythms [1,2].

In the mouse retina, rods (502 nm) are more abundant, while cones constitute 2.7–3% of the photoreceptors [3,4]. In contrast to primates, the murine retina has only two spectral cone types: short (S) cones are sensitive to short wavelengths in the ultraviolet (UV) spectrum (359 nm, short wave (SW)), while long/medium (L/M) cones are sensitive to middle-to-long wavelengths (508 nm, medium wave (MW) and long wave (LW)) [5]. In the mouse retina, topographic separation of different classes of cones has been reported [6]. Variations in retinal topography of S and L/M cones have been observed among different strains (albino and pigmented mice) [7]. In addition, five morphological types of ipRGCs have been identified in mice and rats. These cells have diverse functional roles in non-imaging forming vision and in pattern vision [8,9]. Distinct absorbance spectrum in the different photoreceptor cells is due to apoproteins [10]. These opsins provide specific environment for the absorption of light at particular wavelengths [11]. A protonated Schiff base links opsin and chromophore (retinal), producing a spectral shift from ultraviolet (cromophore: maximal absorption 380 nm) to visible light [12]. However, the S cone cromophore is unprotonated and, consequently, is not capable of such spectral shift (<450 nm) [13].

It has been shown that excessive exposure to visible light can cause toxicity in the vertebrate retina [14]. The degree of damage depends on the level of retinal irradiance, wavelength and exposure duration [15,16]. In this regard, the same visible radiation that activates phototransduction is the responsible for causing damage in photosensitive cells [14].

Phototoxicity related retinal damage has been classified into two groups depending on variables such as the incident wavelength, the exposure time and the cell type affected. Class I takes place after long periods of exposure (days to weeks) to low irradiances and the cells affected are the photoreceptors whose wavelengths are activated [14]. Class II occurs after a short exposure (minutes to hours) to high irradiances of white light and the damage is at level of the retinal pigmented epithelium (RPE) [17]. This effect is also known as "blue-light" damage and has been reported for living animals, either anesthetized [18] or free-running [19].

It has been documented that light causes apoptotic death of photoreceptors and RPE cells [20]. Since light bleaches rhodopsin in the course of this process, it is believed that the main factor of susceptibility to damage is the amount of rhodopsin that can be regenerated after bleaching [18]. During photoactivation of rhodopsin, 11cis retinal becomes all-trans retinal (atRal), which may be condensed with phosphatidylethanolamine (PE) to generate N-retinylidene-phosphatidylethanolamine (N-ret-PE). Both, free atRal and N-ret-PE, are released to the cytosol of outer segments with the help of a member of the ATP binding cassette transporter family (ABC transporters, specifically ABCA4) [21]. In the cytosol, condensation of free atRal with N-ret-PE initiates a process that generates toxic products like A2E (di-retinoid-pyridinium-ethanolamine) [22]. In situations of high illumination, rhodopsin is completely photobleached and free atRal increases above baseline levels. In this scenario, removal of atRal is limited by the ordinary rates of these processes [22].

Current lifestyle relies on artificial illumination that may extend to 16/18 hours per day. Little is known about the levels of light exposure that are safe for the retina [23]. Among the structures that protect the eye from light induced damage are the cornea and the lens, which prevent short wavelengths from reaching the retina. The cornea absorbs wavelengths below 295 nm, while the lens absorbs UV radiation (in the range of 300–400 nm). In addition, these structures (cornea, lens, and even retina) contain chromophores that absorb certain wavelengths, dissipating energy [24].

Blue light (short wavelength) has been shown to be the most harmful to the retina [23,25]. For this reason, many experimental studies have sought to mitigate the effect of blue light radiation on the retinal tissue [26]. It has been reported that in older eyes, due to the yellowish coloration of the crystalline lens, the amount of blue light that reaches the retina decreases [27], giving an extra protection against harmful short wavelengths [28,29]. Cataract extraction and intraocular lens (IOL) implantation could thus increase the amount of blue light that reaches retina. To prevent the deleterious effects of blue light on the retina, the addition of a blue filter to the IOL has been proposed, but advantages of this procedure have not yet been proved clinically [30].

Previous works have shown that the use of blue-blocking filters provides protection to the retina [31,32]. However, the filters used so far absorb blue radiation only partially (30–50%) [33,34]. Furthermore, these filters do not only block blue light but usually block spectra of other wavelengths too [34]. In this work, we have analyzed the structural and functional changes of the retina after exposure to damaging light and the protective effect of a filter that selectively blocks 94% of blue radiation, affecting minimally to other wavelengths (less than 10%). Therefore, we may consider that the effects obtained are genuinely due to blue radiation.

## Materials and methods

### Animals

A total of 18 albino Hds/Win:NMRI mice were used. One-month-old mice were obtained from local providers (JANVIER-Europe, France, www.janvier-europe.com). Before light damage mice were kept in a 12/12 hour light (*ca*. 60 lux) /dark cycle. All animal procedures were carried out in accordance with the ARVO Statement for the Use of Animals in Ophthalmic and Vision Research, the European Community Council (86/609/EEC) guidelines and current Spanish legislation (RD 53/2013). The research and animal experimentation ethics committee of the University of Alcalá approved the protocols applied (permit number OH UAH2016/016)

### Experimental groups and light exposure

Mice were randomly distributed into three study groups (6 animals per group): Group 1: unexposed to damaging light; Group 2: exposed to damaging light without any filter; and Group 3: exposed but protected by a blue blocking filter (ROSCO Company, Roscolux #312, Spain).

In light-exposure experiments, individual animals were placed in a non-acrylic grid cage. Light source was a cold light fluorescent emission lamp (400–820 nm; City Bright International Ltd., Wanchai, China) fixed on the lateral wall of the cage (height 10 cm), at 30 cm of distance from the animal. Mice were exposed to continuous bright light (5,000 lux) for 7 days. The pupils were dilated pharmacologically with a drop of atropin (Colicursí Atropin 1%, Alcan Cusí SA; Spain) applied daily.

### Light composition and light filter parameters

**Fig 1:**
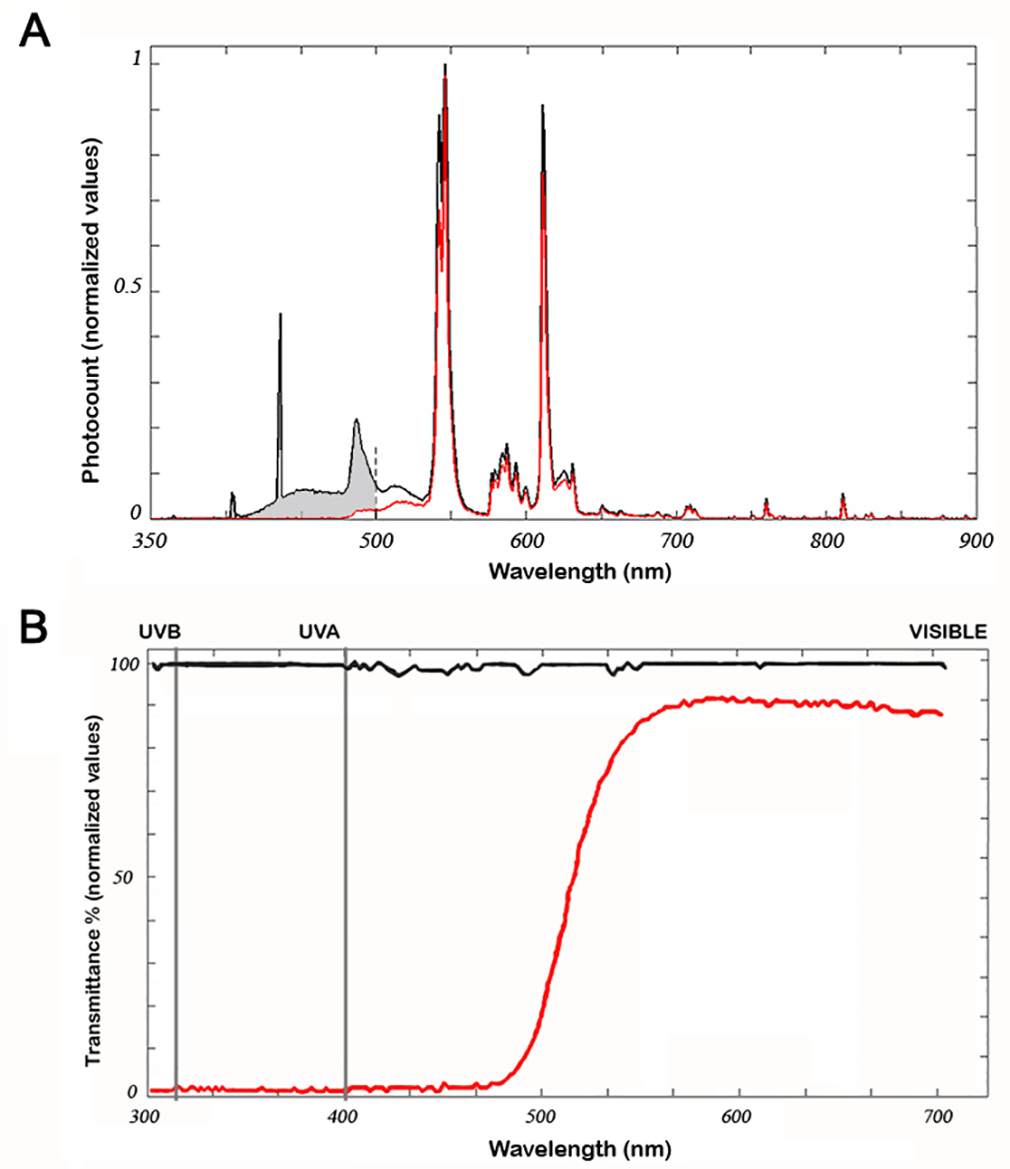
Characterization of the blue-blocking filter. (A) Irradiance of the light source used in phototoxicity experiments in the presence (red trace) or absence (black trace) of the blue-blocking filter. Note that black and red traces are identical for wavelengths longer than 520 nm. The shaded area corresponds to the radiation absorbed by the filter (93.934% of the whole blue radiation). (B) Percent transmittance of the blue-blocking filter: wavelength emissions measured by the Zeiss Humphrey lens analyzer (LA360) without filter (100% value of transmittance; black line) and with the blue-blocking filter (red line).

The spectral irradiance of the fluorescent light source inside the cages, with and without blue-blocking filters, was measured using a spectrometer (USB650 Red Tide Spectrometer; Ocean Optics, USA). The blue-blocking filter absorbed 93.93% of the blue spectrum emitted by our tube lamps (<500 nm), while greater wavelengths were transmitted integrally (Fig 1A). Also, the amount of light of all wavelengths that reached the cage was measured with a spectrophotometer (Zeiss Humphrey LA360 laser, Vision Systems Inc, USA) and expressed as transmittance normalized units (Fig 1B).

### Electroretinography

The electroretinogram (ERG) experiments were carried out with a self-made Ganzfeld Dome illuminated by light emission diodes (RS310-6707; RS-Amidata, Spain). Flash light pulses of 5 ms duration were used for all ERG recordings. PowerLab/4ST (ADInstrumts, United Kingdom) equipment was used for the control of pulse stimulation. ERG recordings were performed in all animals one day before light exposure and eight days after the first ERG. Four animals per group were used for histological experiments (see below), and two animals per group were maintained alive and used for ERG re-test one month after inducing the injury, just to confirm the maintenance of functional photodamage. Full-field electroretinogram was performed following the ISCEV standard protocol (www.iscev.org/standards). Prior to ERG recording, mice were maintained in darkness overnight. Then, mice were anaesthetized under dim red light with an intraperitoneal injection of ketamine (95 mg/kg) and xylazine (5 mg/kg) and kept on a heating pad at 37°C. The pupils were dilated by applying a topical drop of 1% Tropicamide (Colicursí Tropicamida, Alcan Cusí SA, Barcelona, Spain). A topical drop of 2% Methocel (Ciba Vision AG, 8442 Hetlingen, Switzerland) was instilled in each eye immediately before placing the corneal electrode. Anesthetized animals were placed on a Faraday cage and experiments were performed sequentially in scotopic and photopic conditions. Flash-induced ERG responses were recorded from the right eye in response to light stimuli. Retinal responses mediated by rod photoreceptors (Rod-driven Response) were recorded under dark adaptation to light flashes of -2 log cd·s·m^-2^. Rod and cone mediated responses (Mixed rod-cone Responses) were recorded under dark adaptation in response to light flashes of 0.48 log cd·s·m^-2^. Oscillatory potentials (OP) were isolated from the mixed response by band pass filtering between 100–10,000 Hz. Under scotopic conditions, five to ten light responses were averaged for each ERG trace; flash intervals were set at 30 seconds. Then, the animals were light-adapted for 10 minutes under white rod-suppressing background light (100 cd·m^-2^) and photopic responses were recorded. Responses mediated by cone photoreceptors (Cone-driven Response) were recorded under photopic conditions in response to light flashes of 2 log cd·s·m^-2^. Under photopic conditions thirty to sixty responses were averaged and flash intervals were set at 1 second. ERG signals were amplified and band filtered between 1 and 1,000 Hz with a Grass amplifier (CP511 AC amplifier, Grass instruments, Quinay, MA, USA). Electrical signals were digitalized at 20 KHz with Power Lab data acquisition board (Power Lab 4ST). Electrical potential changes were recorded with a fixed corneal lens electrode (Burian-Allen electrode, Hansen Ophtalmic Development Lab Coralville, IA, USA), a reference electrode located inside the mouth and a ground electrode located on the tail. Calibration of the light stimuli was performed with a digital photometer (GO 4068 Gossen Mavo-monitor USB, Gossen Corporation, Milwaukee, USA).

**Fig 2:**
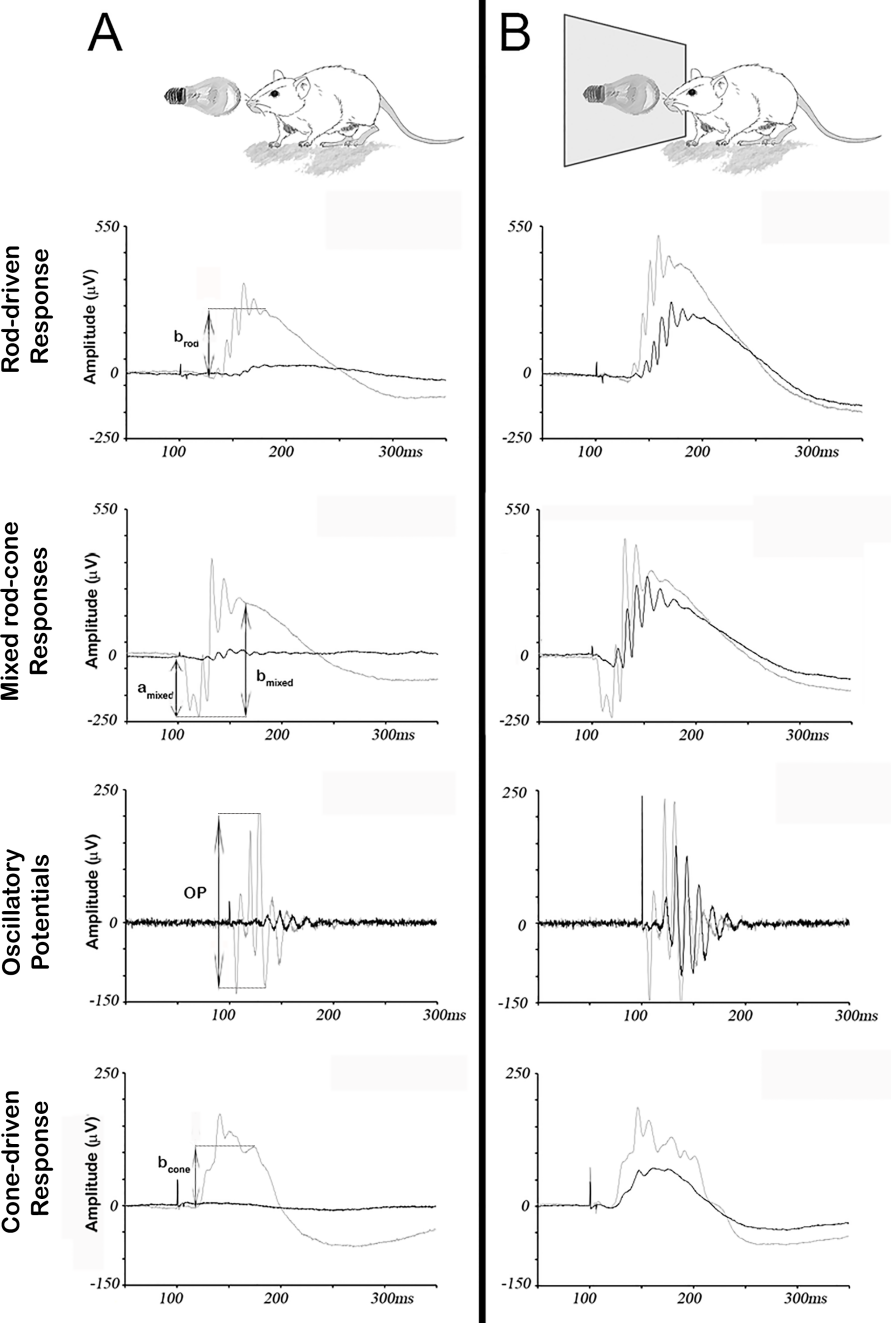
Protective effect of the blue-blocking filter on retinal response to damaging light. Representative ERG traces from unprotected (A) and protected (B) groups of mice, obtained before (grey traces) and after (black traces) exposure to white light (5,000 lux) for seven days. Arrows indicate the amplitudes measured for each different ERG wave. In the unprotected group (A), amplitudes of scotopic (rod-driven, mixed rod-cone and Oscillatory Potentials) and photopic (cone-driven) parameters are strongly reduced upon light-induced damage, while in the protected group (B), this reduction is attenuated.

ERG wave amplitudes were measured for every recording and averaged from the six animals of each experimental group. The amplitude of the positive deflection of the Rod-driven Response (namely *b*_*rod*_) was measured from the baseline to the peak of the positive deflection. The amplitude of the negative deflection of the Mixed rod-cone Response (namely *a*_*mixed*_) was measured from the baseline to the trough of the negative deflection. The amplitude of the positive deflection of the Mixed rod-cone Response (namely *b*_*mixed*_) was measured from the trough of the negative deflection to the peak of the positive deflection (excluding the oscillatory potentials). The amplitude of the OP was measured from peak to peak. The amplitude of the positive deflection of the Cone-driven Response (namely *b*_*cone*_) was measured from the baseline to the peak of the positive deflection. Arrows in Fig 2 show the measurement criteria for the amplitudes of the ERG responses. The retinal origin of the different ERG components was elucidated in previous works [35] and we used the nomenclature recommended by ISCEV [36,37].

### Retinal histology

A total of 12 animals (4 per group) were sacrificed after the second ERG recording by cervical dislocation and both eyes were enucleated and immersed in 4% (w/v) paraformaldehyde for 2 hours at room temperature. After removing the cornea and lens, the eyes were immersed in the same fixative solution for one more hour. Then the eyes were sequentially cryoprotected in 20% (1 hour), 30% (1 hour) and 40% (overnight) sucrose at 4°C. Eyecups were then embedded in tissue tek, oriented and frozen in liquid nitrogen. An average of 15 dorsal-ventral retinal sections, 12-μm-thick, were obtained with a cryostat (Leica CM3000 Cryostat, Leica Biosystems, Nussloch, Germany) from each eye.

After washing with 0.1M PBS (phosphate-buffered saline, pH 7,4, SIGMA-ALDRICH, St Louis, USA), retinal sections were preincubated for 1 hour and 30 minutes with 0.1M PBS containing 2% (w/v) bovine serum albumin (BSA) and 0.02% (v/v) Triton X-100 (PBS-Tx). A total of six retinal sections per animal were immunostained by incubation in PBS-Tx containing goat polyclonal anti-Red/Green opsin (1:200 dilution; #AB5405; Merck MILLIPORE, Billerica, MA, USA) for 14–15 hours at room temperature. After washing, sections were incubated with Cy3-conjugated secondary antibodies (1:200 dilution; Jackson, West Grove, PA, USA) for 1 hour. Negative controls omitting the primary or secondary antibody were included. In addition, nuclei were stained with 4', 6-diamidino-2-phenylindole dihydrochloride (DAPI) at 5 mg/ml (#10 236 276 001; Sigma Aldrich). Sections were mounted with a mixture (0.42% glycine, 0.021% NaOH, 0.51% NaCl, 0.03% sodium azide, 5% N-propylgalate, 70% glycerine and distilled water) for fading protection purposes.

Detection of apoptotic nuclei was accomplished by the Terminal-deoxynucleotidyl transferase dUTP nick end labeling (TUNEL technique). A total of six retinal sections per animal were fixed in ethanol and acetic acid (2:1) at -32°C for 5 minutes. Then, sections were rinsed with 0.1M PBS and immersed in 0.1M PBS with 0.2% (v/v) Triton X-100 and 0.1% (w/v) sodium citrate for 15 minutes. After washing, sections were immersed in TUNEL buffer (30mM Tris, 140 mM sodium cacodylate, 1mM cobalt chloride, 0,3% Tritón X-100 and distilled water) for 30 minutes and then they were incubated 1 hour and 30 minutes with the same buffer containing terminal deoxynucleotidyl transferase (#11767305001; TdT; Roche; Switzerland) (800 U/ml) and dUTP (#11093070910; Roche) (1 μM) at 37°C. The reaction was terminated by addition of sodium chloride and sodium citrate buffer followed by rinsing with 0.1 M PBS. After staining with DAPI, all sections were washed with PBS three times and cover-slipped with mounting medium. Microscope fluorescent images were obtained with a LEICA TCS-SP5 laser confocal microscope (Leica Instruments, Wetzlar, Germany).

### Photoreceptor count

Cell count was performed on DAPI stained sagittal retinal sections. A total of six sections per animal were averaged for cell counts. In each section, four regions of interest (200 μm in length each) [38], located along the dorsal-ventral axis at four different eccentricities: 30° dorsal (Z2) and ventral (Z3), and 60° dorsal (Z1) and ventral (Z4) from the optic nerve head, were selected. Photoreceptor nuclei in each region of interest were counted manually on the six retinal sections from the four animals of the three experimental groups (Table 1).

**Table 1:** Photoreceptor cell counts in the different experimental groups (mean ± SD).

	Unexposed	Protected	Unprotected
Location			
**Z1**	541 ± 135	336 ± 64	209 ± 97
**Z2**	544 ± 136	314 ± 73	104 ± 67
**Z3**	524 ± 131	367 ± 71	208 ± 73
**Z4**	605 ± 151	403 ± 86	320 ± 28
**Total**	2214 ± 552	1421 ± 263	841 ± 214

### Statistical analysis

The amplitudes of the different ERG waves were compared in protected and unprotected animal groups using unpaired Student´s *t*-test with Welch correction. Changes in global number of surviving cells were studied between different groups by two-way ANOVA (*two-tailed*). Differences between retinal areas within the same experimental group were studied by two-way ANOVA (*two-tailed*) followed by Tukey’s posthoc test. Statistical analyses were performed with GraphPad Prism version 5.00 for Windows (Graph Pad Software, San Diego, CA, USA, www.graphpad.com).

## Results

In a series of preliminary experiments, albino mice were exposed to white light (5,000 lux) for 2, 3 or 5 days (data not shown). All these animals suffered a decrease in the amplitude of their ERG waves. However, a functional recovery was observed in those animals in which light exposure lasted 5 days or less. Then, we decided to expose animals to light for 7 days. No functional recovery was observed for such exposure time.

### Electroretinogram recordings

**Fig 3:**
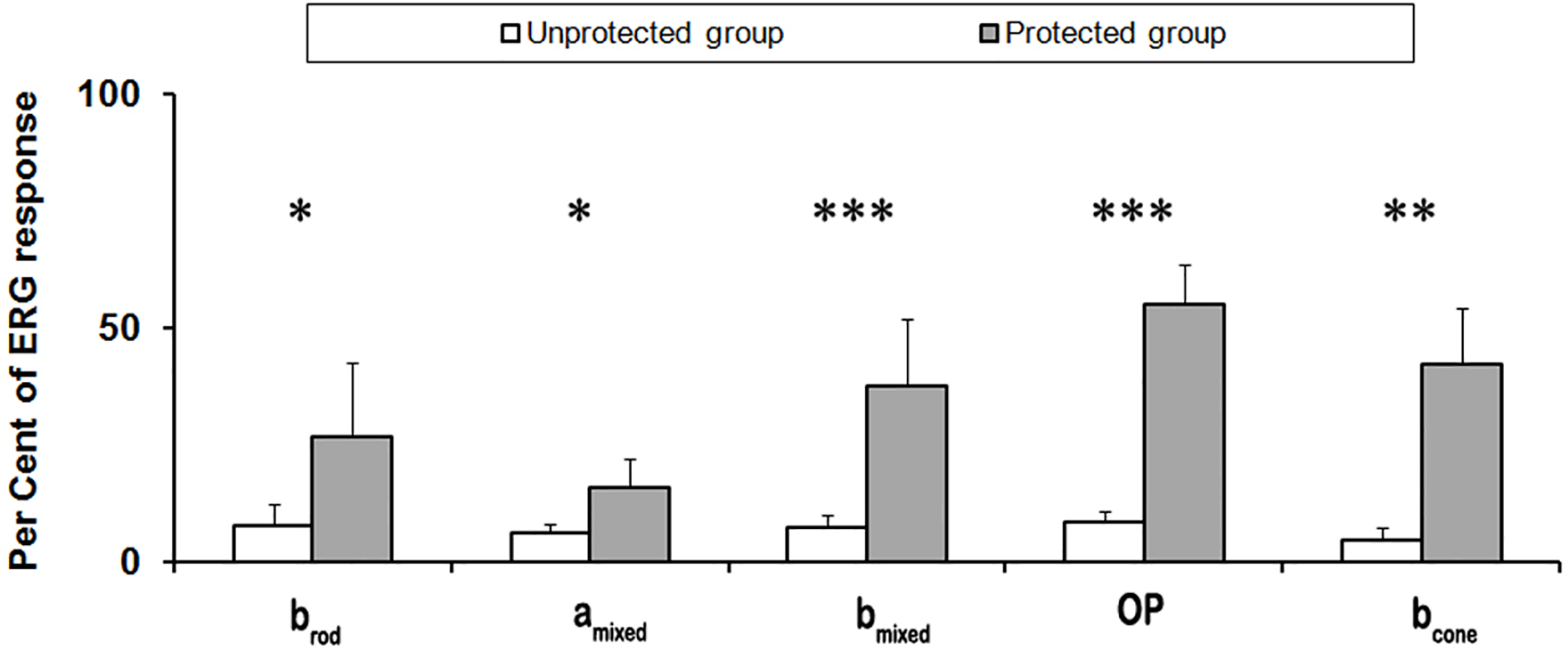
Effect of the blue-blocking filter on ERG wave amplitudes. Percentages of ERG wave amplitudes in unprotected and protected groups after exposure to white light (5,000 lux for seven days; 100% response corresponds to averaged ERG wave amplitudes before the exposure). Statistically significant differences were observed in all measured ERG parameters Data in each group were averaged from a total of 6 animals (mean ± SD). Asterisks indicate significant differences using unpaired *Student´s t-test* (*p<0.05, **p<0.01, ***p<0.001).

To evaluate the potential protective effect of a blue-blocking filter on visual responses, two groups of mice were exposed to 5,000 lux of fluorescent light for 7 days. In one of the groups, a filter that absorbed 94% of the blue spectrum (<500 nm) covered permanently the light source. Following retinal damage, scotopic and photopic visual responses were evaluated by full-field ERG and compared to equivalent recordings performed before light exposure. Amplitudes for five different ERG waves (*b*_*rod*_, *a*_*mixed*_, *b*_mixed_, oscillatory potentials (OP) and *b*_cone_) were measured and averaged from both groups, unprotected and protected (Fig 2 and S1 Fig). In unprotected mice, ERG wave amplitudes were reduced approximately 90% (Figs 2A and 3). However, under the effect of the blue-blocking filter, the decrease was only about 40% (Figs 2B and 3). As shown in Fig 3, the differences between unprotected and protected animals were statistically significant for all ERG wave amplitudes studied. Therefore, despite protection of retinal function was not complete, the blue-blocking filter partially prevented light-induced damage to the retina.

### Photoreceptor cell count and morphology after light exposure

**Fig 4:**
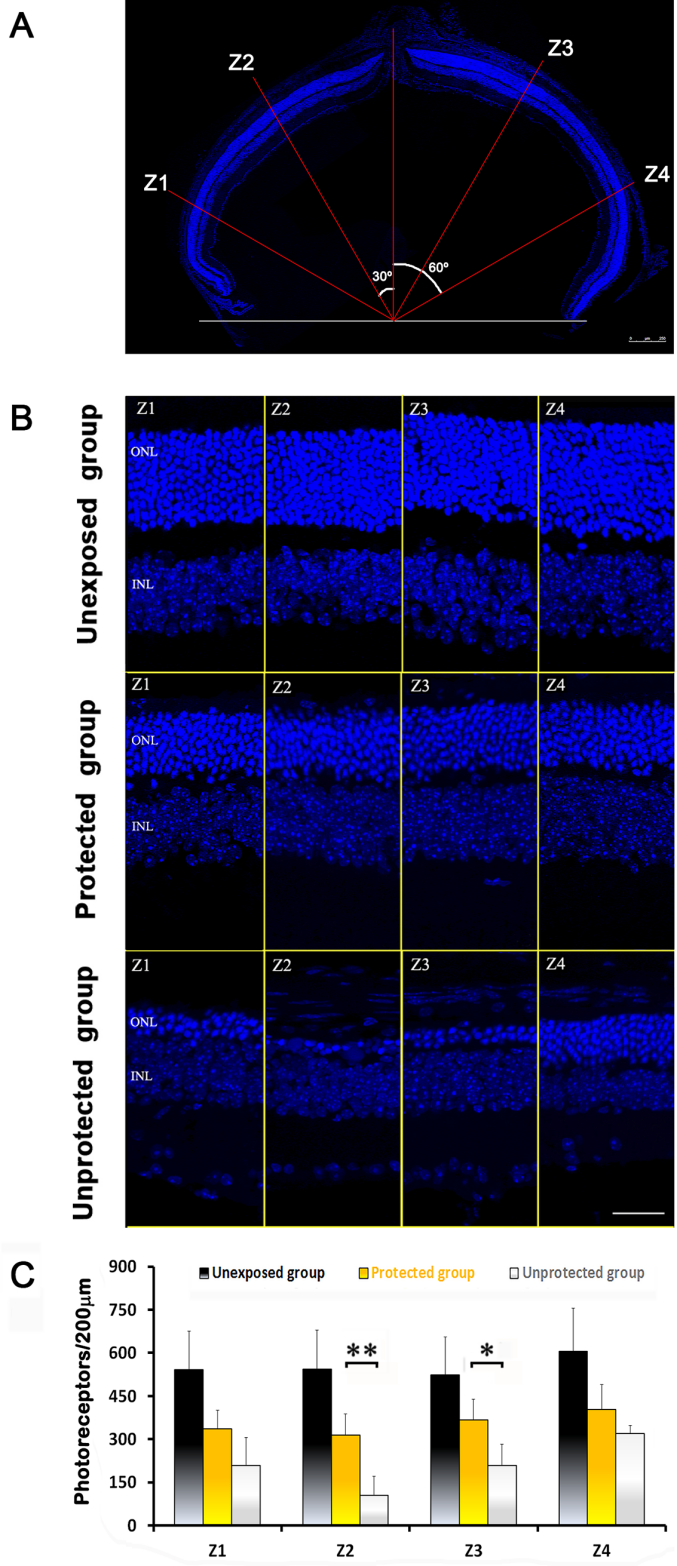
Number of surviving photoreceptors under different experimental lighting conditions. (A) Photoreceptor survival was analyzed in cryosections at four retinal eccentricities: Z1, 60° dorsal from the optic nerve head (ONH); Z2, 30° dorsal from the ONH; Z3, 30° ventral from the ONH; and Z4, 60° ventral from the ONH. Scale bar 250 μm. (B) Representative images of retinal sections stained with DAPI from the three experimental groups. ONL: outer nuclear layer, INL: inner nuclear layer. Scale bar: 80 μm. (C) Averaged number of photoreceptors (mean ± SD; n = 4) at the different eccentricities. Statistically significant differences were observed between the unexposed group and the other two for all retinal eccentricities (p<0.01, *two way ANOVA*) and between the unprotected and the protected groups for Z2 and Z3 eccentricities (*p<0.05, **p<0.01, respectively; *two way ANOVA*).

Following assessment of visual function, we wanted to correlate our findings with the number of surviving photoreceptors in the ONL. Retinal sections obtained in the dorsal-ventral axis were stained with the fluorescent dye DAPI to label nuclei. In each retinal section four regions of interest, at different eccentricities, were selected (Fig 4A) and the total number of photoreceptor nuclei was counted in a 200-μm-long sampling region (Fig 4B). For every retinal location, the number of photoreceptors in the unexposed group of mice was significantly higher than in the unprotected or the protected groups (p<0.01, *two way ANOVA*) (Fig 4B and 4C). In addition, statistically significant differences were found between the protected and the unprotected groups for retinal locations Z2 (p<0.01) and Z3 (p<0.05) (Fig 4C, Table 1 and S2 Fig), which correlated with the protective effect of the blue-blocking filter, already seen by ERG.

In unexposed and protected groups, the number of photoreceptor nuclei was similar between different sampling locations within the same retinal section, i.e. statistically significant differences were not found using *one way ANOVA*. On the contrary, the number of photoreceptor nuclei between different sampling locations was variable in the unprotected group of mice. Specifically, significant differences were found in retinal locations Z2 and Z4 (p<0.01, *one way ANOVA;* Tukey’s multiple comparison post-test) (Table 1).

**Fig 5:**
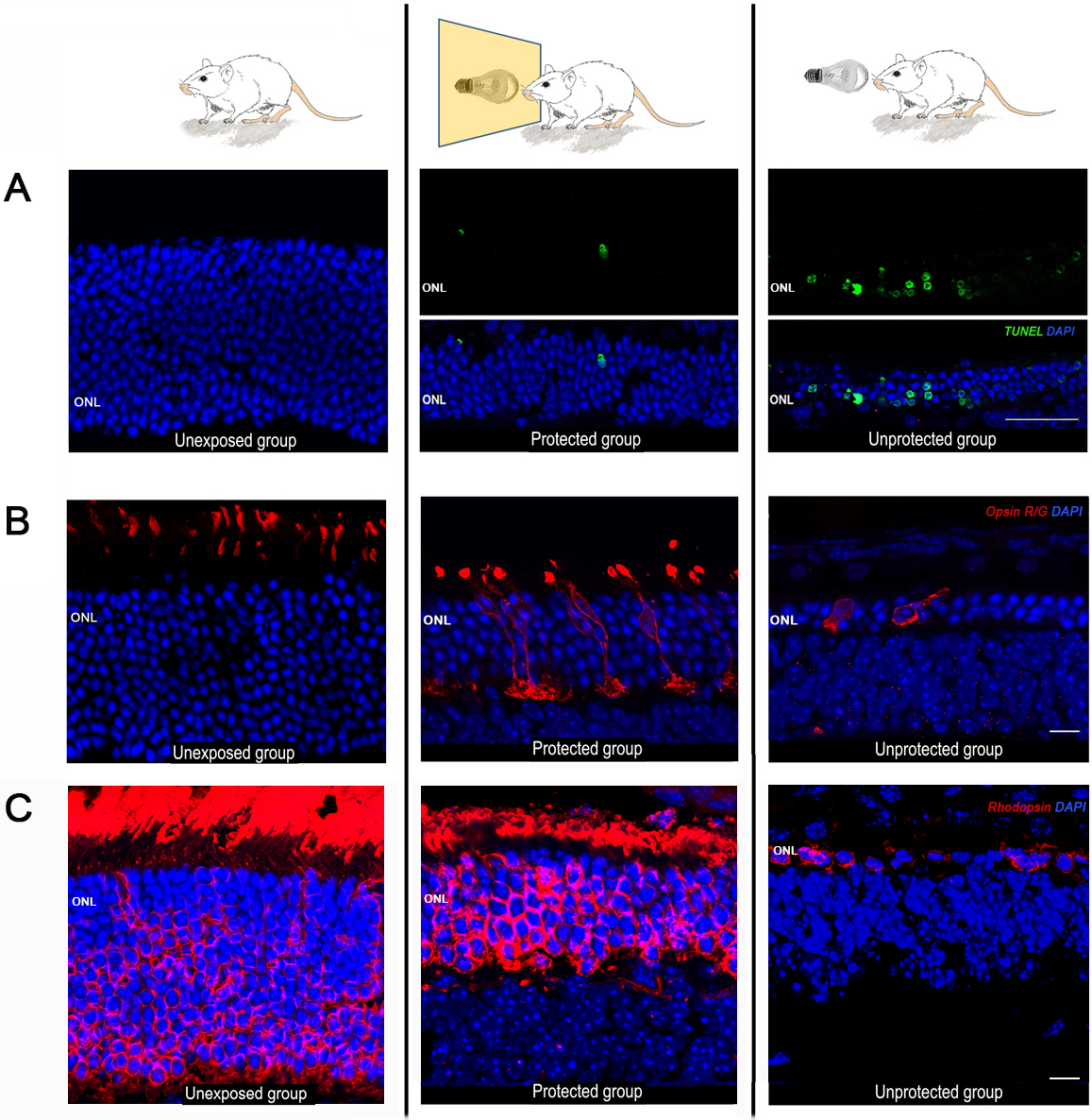
Photoreceptor cell death and protective effect of the blue-blocking filter on cone photoreceptors. Representative photomicrographs of retinal sections (eccentricity Z2, 30° dorsal from the ONH) obtained from the three experimental groups: unexposed (left panel), protected (middle panel) and unprotected (right panel). (A) Photoreceptor cell death was revealed by the TUNEL technique (green fluorescence). Unprotected animals showed an intense labeling at the ONL, while little or no labeling was observed in protected and unexposed animals, respectively. Nuclei were counterstained with DAPI (blue fluorescence). Scale bar: 50 μm. (B) Immunostaining of cone photoreceptors with red/green opsin antibodies (red fluorescence). Scale bar: 10μm. (C) Immunostaining of rod photoreceptors with rhodopsin antibodies (red fluorescence) Gain settings were maintained at the same level for all experimental groups. Scale bar: 7 μm.

Cell death was studied by TUNEL labeling of retinal sections. While in unexposed animals no labeling was observed, in mice exposed to damaging light TUNEL+ cells could be detected in the ONL. However, the amount of apoptotic nuclei in unprotected retinas was larger than in the protected group (Fig 5A).

Appropriate morphology of photoreceptors and normal compartmentalization of visual pigments are signs of retinal wellbeing. Thus, the shielding effect of the blue-blocking filter, evidenced by ERG recordings, should correlate with an improved morphology of rods and cones in the protected group of mice. To evaluate photoreceptor morphological changes following light-induced damage, as well as the degree of protection conferred by the blue filter, retinal sections were immunostained with antibodies against red/green opsin, to label single cones, and rhodopsin, to visualize rod outer segments and somas (Fig 5B and 5C). In unexposed retinas immunostained with red/green opsin, only cone outer segments were labeled, as expected (Fig 5B, left panel). In the protected group, however, the labeling extended to the somas, axons and axon terminals, suggesting that cone opsin was being redistributed along cell compartments (Fig 5B, middle panel). Also, cone outer segments were shorter than in the unexposed group. In the unprotected group cones showed an abnormal morphology, lacking outer segments, axons and axon terminals (Fig 5B, right panel). Rhodopsin expression was analyzed in every group as well (Fig 5C). The unexposed group showed strong labeling of rod outer segments and somas (Fig 5C, left panel). A similar pattern was observed in the protected group, but the size of the outer segments and the number of nuclei rows in the ONL was diminished (Fig 5C, middle panel). The unprotected group showed a huge decrease in the number of rod somas and a lack of outer segments, mainly at Z2 location (Fig 5C, right panel). Therefore, light-induced decrease in visual function correlated with photoreceptor morphological changes and apoptosis, which were attenuated in the presence of a blue-blocking filter.

## Discussion

The aim of this work was to test the protective capacity of a specific blue filter against the effects of damaging light. To meet this purpose, retinas were exposed to a constant and intense light and, consequently, suffered a significant decrease in the number of photoreceptors and the amplitude of their functional recordings. However, when the same exposure to light took place through a blue-blocking filter, the decrement in the number of photoreceptors was significantly lower in central areas of the retina and the loss of functional response was attenuated.

Before analyzing the protective effect of the blue-blocking filter, it was necessary to know the exact amount of light that was being removed from the spectrum. In order to ascertain this, spectrometric measurements of the same light source used afterward to induce retinal damage, were carried out in the presence or absence of the filter. We found that the filter used in this study absorbed up to 93% of light below 500 nm and, at the same time, did not alter other components of the spectrum above 530 nm. Therefore, we were able to characterize, in a rather accurate way, structural and functional retinal damage caused by blue light. Similar studies recently published, like the one performed by Narimatsu and colleagues [34], were less precise in terms of the characterization of the protective effects of blue-blocking filters, given that their transmittance data showed that a large percentage of the emitted light below 500 nm passed through the blocking filter (blue-light blockade was 24% or 40%, depending on the filter used). In addition, the transmittance decrease of non-blue wavelengths, due to the use of filters, was disregarded. Therefore, in the present study we provide additional reliable data regarding the efficacy of blue-blocking filters that agree with and round up what has been published before.

Following exposure to damaging light, a decrease in functional responses has been reported. Complete disappearance of the a-wave means that photoreceptors suffer a very aggressive damage, while a decreased b-wave suggests that rod and cone pathways are altered. However, when mice are housed in the same light conditions but with a blue-blocking filter, retinal responses are partially maintained [39]. Some studies have looked for differences between light exposure with and without filters, but not changes within the same groups after light exposure. Our results show pre and post-stress differences for each light condition. This is especially necessary, and not always done, if we take into account the great variability that may exist between mice of the same strains.

Some phototoxicity protocols require a long exposure to low intensities of light [40], while others require a shorter exposure to higher intensities [39,41]. Additionally, some studies have reported to induce photochemical damage through exposure to moderate light for a short time although this damage was temporary when the exposure did not exceed 12 hours [42]. However, some other studies report transient damage as well for exposures longer than 12 hours [40]. In contrast, functionality could not be recovered after a long exposure in our hands. A possible explanation could lie in the use of a higher light intensity. Differences between all these studies could be also attributed to the use of different species or strains and to the housing conditions (plastic cages could cause an increase in temperature).

Functional impairment has a direct correlation with structural alterations in the retina. The low expression of cone opsin in unprotected animals may justify the lack of cone response observed in the ERG. Differences in the number of photoreceptors between protected and unprotected groups were significant in central areas of the outer nuclear layer, mainly in Z2 (dorsal retina). This was in agreement with other works that tested the protective effect of different antioxidants and growth factors in the light-induced retinal damage model and reported the strongest protective effect in the dorsal-temporal retina [41,43,44]. Of note, the smallest density of blue cones can be found in this particular area [7,45]. Therefore, it is reasonable to think that photoreceptors lacking chromophores for short wavelengths show increased sensitivity to light-induced damage when the blue light is involved. Interestingly, when retina is exposed for a long time to non-blue wavelenghts, all areas are affected similarly.

Although the blue-blocking filter promoted photoreceptor survival, this protection was incomplete. Remaining damage might be attributed to the residual blue radiation (in the range of 400 to 500 nm) passing through the filter. Given that the filter used in our experiments did not avoid radiation between 500 and 580 nm, another possible explanation for the residual damage to the retina would imply green light, which is potentially harmful [46]. However, it has been reported that green light does not seem to induce photoreceptor apoptosis after short periods of exposure [18].

The residual damage in the protected group was evidenced by the presence of TUNEL positive cells, more previously described [47,48], although this group showed a lower number of TUNEL+ profiles than the non-protected one. In high light exposure protocols, death mechanisms occur despite the protection system used [41].

It has been reported that a deprivation of light for 9 months, after suffering a 50% reduction in functional responses caused by photostress, allowed a full recovery [40]. For this reason, it is reasonable to think that although our filter does not avoid damage completely, injury could be mitigated enough for getting a full recovery later.

After photostress, cones and rods suffer severe modifications before dying by apoptosis. Such morphological changes occur throughout different phases, involving a series of metabolic processes whose ultimate stage is the demise of the cell [49] Thus, destruction of photoreceptors explains the irreversible loss of retinal function. The use of filters allows the survival of a large number of photoreceptors, but does not completely prevent the development of morphological and functional alterations in the retina. One of these modifications is the change in the distribution of opsins, which may vary depending on cell types [50]. The cause may be in the wavelengths at which chromophores are excited. While for rods and SW cones is 500 nm or less, for L/M cones is 508 nm. Thereby, the filter would prevent the passage of the lowest wavelengths and allow excitation of most L/M cones.

Different opsins are composed of an apoprotein and a specific chromophore. Since the synthesis of new apoprotein is dependent on the excitation of the chromophore [51], an elevated excitation of the chromophore will produce a greater synthesis of apoprotein. This would explain why L/M cones are able to synthesize more apoproteins than rods.

The increment of rhodopsin bleaching products (atRal) produces retinal photodamage, as previously reported [52]. Furthermore, if light exposure is high enough, photobleaching (high levels of atRal) can cause a permanent damage [41]. Physiologically, atRal within Metharhodopsin II (a metastable state of rhodopsin) is cleared by natural mechanisms (ABCA4 transporter and enzymatic reduction by retinal DH) in a slow process [22,53]. However, when rhodopsin regeneration is faster than atRal reduction, free atRal may increase [51]. It is known that free atRal (a toxic aldehyde) is a powerful photosensitizer when exposed to UVA or blue light of high intensity and long duration [21,54,55], comparable to our experimental conditions. It has been shown atRal is greatly released through three different mechanisms [21,54,55]. The first one is due to the bleaching of all rhodopsin at MII. The second implies the presence of all wavelengths (including blue visible light), which allows rhodopsin photoregeneration [53] and inactivation of ABCA4 transport [54], both leading to further increase in atRal accumulation. The third, photoreversal, implies the re-isomerization of the retinal chromophore after absortion of additional photons by other ways. Free atRal causes photoxidative damage to the retina through singlet oxygen [56] and as a precursor of A2E.

**Fig 6:**
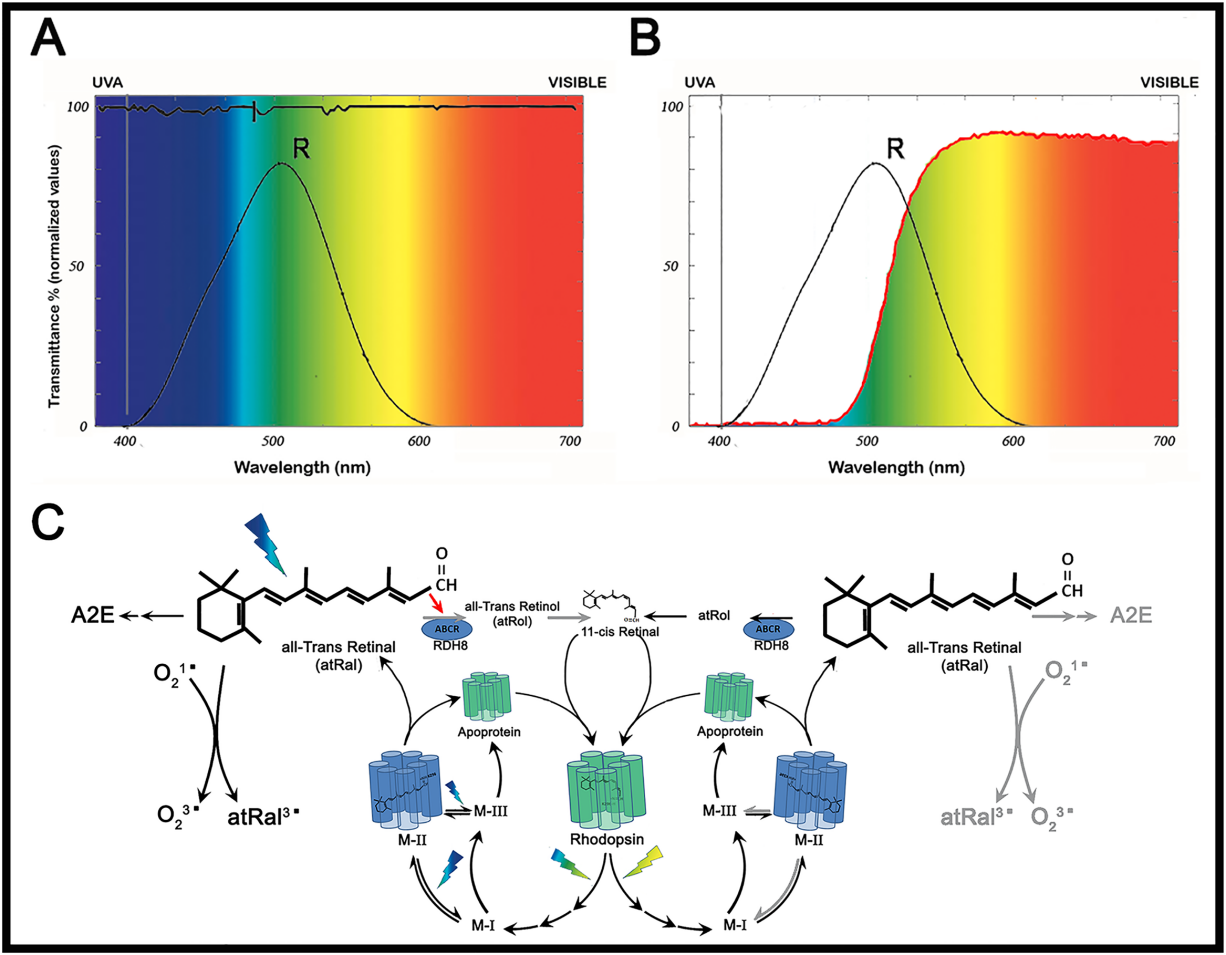
Excitation and metabolic cycle of rhodopsin depending on the use of the blue-blocking filter. (A) In the presence of light of all wavelengths, rhodopsin absorption spectrum falls below the black curve. (B) When a blue-blocking filter is used, the spectrum is only possible in the space below the black curve limited by the red line. (C) All-trans Retinal (atRal) is generated after rhodopsin excitation. This may follow two different pathways depending on the excitation wavelength: after a blue-green-yellow flash (400 to 600 nm) or after a green-yellow flash (500 to 600 nm). Both pathways are represented to the right and left of the rhodopsin molecule, respectively. In high and continuous light exposure, total bleaching of rhodopsin takes place and much toxic atRal is accumulated. Then, atRal is slowly recycled to MII in a process that involves blue light (photoreversal). Blue light is also implied in MIII storage increase. The huge atRal accumulation and its photoactivation by blue light have important implications for photoreceptors, like the genesis of photoreactive metabolites (A2E and lipofuscin). Also, in the presence of molecular oxygen, photo-oxidative reactions are initiated, as well as blocking of its conveyor ABCA4 and subsequent action of retinal dehydrogenase (in rods, RDH8) (red arrow). Conversely, in the absence of blue light all these processes disappear or are strongly attenuated (right side of the drawing). Gray arrows indicate a decreased effect or a blockade.

After light exposure there is a retinal damage. However, rhodopsin stimulation does not disappear completely, as evidenced by functional responses (Fig 6A and 6B). Illumination through a blue-blocking filter would possibly cause a decrease in atRal photoexcitation due to the lack of blue light [22,55]. In addition, inhibition of ABCA would not occur, enabling the recycling of atRal [21], and the activation pathways triggered by toxic aldehydes would be attenuated as a consequence of blue light removal. However, even in these conditions, remaining excitation could be due to green light that has the ability to bleach rhodopsin without causing photoreversal [53] (Fig 6C).

Wavelengths that can activate rhodopsin range from 400 nm to almost 600 nm [5]. We can distinguish two parts in the spectrum. One consists on the "non-blue" (above 500 nm) wavelengths that excite rhodopsin and generate toxic waste, but do not cause retinal degeneration. The other part of the spectrum, below 500 nm, causes retinal degeneration in addition to toxic waste. This degeneration seems to affect primarily retinal cells capable of absorbing photons of these wavelengths. Rhodopsin and its sub-products of excitation seem to have a major role in retinal damage.

The relationship among blue light, generated toxic products (atRal, A2E, lipofuscin) and degenerative diseases such as age-related macular degeneration, Stargardt’s disease and others [54,57,58], makes it necessary to control the amount of radiation that the human retina receives. These reasons are especially important given our current lifestyles, both from a technological point of view (the use of blue LED on mobile terminals, screens, etc.), as well as from an occupational perspective (night shifts with high exposure to blue lights).

## Conclusions

In summary, retinal photodamage caused by a conventional light source (cold) can become chronic if exposure is high and long enough. In contrast, the use of blue-blocking filters can significantly alleviate the functional loss of retinal photosensitive cells. Therefore, these filters might be an effective mechanism to protect us from ocular pathologies.

## Supporting information

S1 Fig Values of ERG wave amplitudes. Data of unprotected and protected groups after exposure to white light. Fig 3 and the subsequent statistical analysis were done with these data.
(DOCX)

S2 Fig Number of surviving photoreceptors following exposure to white light. Photoreceptor survival in unexposed, protected and exposed retinas, after light-induced damage. Cell counting was performed at four different retinal eccentricities. Fig 4C and the subsequent statistical analysis were done with these data.
(DOCX)

## Abbreviations

A2E: di-retinoid-pyridinium-ethanolamine
ABCA4: ABC binding cassette transporter 4
ARVO: Association for Research in Vision and Ophthalmology
atRal: all-trans retinal
BSA: bovine serum albumin
DAPI: 4' 6-diamidino-2-phenylindole dihydrochloride
dUTP: deoxyuridine triphosphate
ERG: electroretinographical
IOL: intraocular lens
ipRGCs: intrinsically-photosentitive retinal ganglion cells
ISCEV: International Society for Clinical Electrophysiology of Vision
L/M: Long/Medium
LED: light emission diode
LW: Long Wave
MW: Medium Wave
NMRI: Naval Medical Research Institute
N-ret-PE: N-retinylidene-phosphatidylethanolamine
ONL: outer nuclear layer
OP: oscillatory potentials
PBS: phosphate buffered saline
PE: phosphatidylethanolamine
RPE: retinal pigmented epithelium
S: Short
SW: Short Wave
TdT: deoxynucleotidyl transferase
TUNEL: Terminal-deoxynucleotidyl transferase dUTP nick end labeling
Tx: triton X-100
UV: Ultraviolet.
