# Supplementary material for: Removal of the blue component of light significantly decreases retinal damage after high intensity exposure
Source: PLoS One. 2018 Mar 15;13(3):e0194218. doi: 10.1371/journal.pone.0194218 (PMC5854379; doi:10.1371/journal.pone.0194218)
Supplement: S1 Fig — Data of unprotected and protected groups after exposure to white light. Fig 3 and the subsequent statistical analysis were done with these data. (DOCX) [file pone.0194218.s001.docx]

**S1Fig.**

| **Protected group (6)** | | | | | |
| --- | --- | --- | --- | --- | --- |
|  |  |  |  |  |  |
| **Mouse** | **Rod** | **Mixed a** | **Mixed b** | **OP** | **Cone** |
| **11020** | 51,18 | 25,45 | 44,94 | 53,31 | 36,33 |
| **11059** | 27,15 | 12,42 | 33,21 | 67,89 | 29,89 |
| **11060** | 11,16 | 15,24 | 15,29 | 52,46 | 37,14 |
| **11055** | 10,77 | 16,87 | 47,17 | 51,17 | 40,68 |
| **11058** | 39,20 | 12,23 | 46,33 | 63,27 | 34,54 |
| **11061** | 20,30 | 13,24 | 36,56 | 52,36 | 56,35 |
| **Mean** | **26,63** | **15,91** | **37,25** | **56,74** | **39,15** |
| **Stand desviat** | **16,07** | **5,00** | **12,16** | **7,03** | **9,13** |
|  |  |  |  |  |  |
|  |  |  |  |  |  |
|  |  |  |  |  |  |
| **Unprotected group (6)** | | | | | |
|  |  |  |  |  |  |
| **Mouse** | **Rod** | **Mixed a** | **Mixed b** | **OP** | **Cone** |
| **11017** | 10,18 | 6,36 | 4,83 | 9,45 | 2,43 |
| **11035** | 4,60 | 3,63 | 3,49 | 5,78 | 2,96 |
| **11053** | 9,24 | 8,47 | 7,80 | 7,85 | 3,22 |
| **11054** | 4,34 | 5,33 | 5,00 | 9,36 | 11,77 |
| **11037** | 11,56 | 8,65 | 10,65 | 12,32 | 5,04 |
| **11052** | 3,45 | 5,46 | 9,52 | 8,24 | 1,39 |
| **Mean** | **7,23** | **6,32** | **6,88** | **8,83** | **4,47** |
| **Stand desviat** | **3,49** | **1,95** | **2,87** | **2,17** | **3,77** |

Values of all mice in percentages of ERG wave amplitudes. We show data of unprotected and protected groups after exposure to white light. Fig 3 and the subsequent statistical analysis were done with these data.
