# Supplementary material for: Removal of the blue component of light significantly decreases retinal damage after high intensity exposure
Source: PLoS One. 2018 Mar 15;13(3):e0194218. doi: 10.1371/journal.pone.0194218 (PMC5854379; doi:10.1371/journal.pone.0194218)
Supplement: S2 Fig — Photoreceptor survival in unexposed, protected and exposed retinas, after light-induced damage. Cell counting was performed at four different retinal eccentricities. Fig 4C and the subsequent statistical analysis were done with these data. (DOCX) [file pone.0194218.s002.docx]

**S2 Fig.**

| **Protected group** | | | | | |
| --- | --- | --- | --- | --- | --- |
| **Mouse** | **Total** | **Z1** | **Z2** | **Z3** | **Z4** |
| **11020** | 1691 | 394 | 379 | 400 | 518 |
| **11058** | 1542 | 354 | 320 | 452 | 416 |
| **11059** | 1374 | 354 | 347 | 311 | 362 |
| **11060** | 1078 | 244 | 211 | 305 | 318 |
| **Mean** | **1421,25** | **336,50** | **314,25** | **367,00** | **403,50** |
| **Stand desviat** | **262,93** | **64,49** | **72,94** | **71,40** | **86,21** |
|  |  |  |  |  |  |
| **Unprotected group** | | | | | |
| **Mouse** | **Total** | **Z1** | **Z2** | **Z3** | **Z4** |
| **11017** | 595 | 144 | 21 | 111 | 319 |
| **11052** | 729 | 109 | 84 | 205 | 331 |
| **11053** | 1030 | 272 | 176 | 234 | 348 |
| **11054** | 1010 | 310 | 134 | 284 | 282 |
| **Mean** | **841,00** | **208,75** | **103,75** | **208,50** | **320,00** |
| **Stand desviat** | **213,96** | **97,29** | **66,77** | **72,73** | **27,99** |
|  |  |  |  |  |  |
| **Unexposed group** | | | | | |
| **Mouse** | **Total** | **Z1** | **Z2** | **Z3** | **Z4** |
| **12011** | 2469 | **592** | 601 | 604 | 672 |
| **12013** | 1397 | **343** | 345 | 329 | 380 |
| **12016** | 2599 | **645** | 651 | 603 | 700 |
| **12017** | 2393 | **587** | 578 | 559 | 669 |
| **Mean** | **2214,50** | **541,75** | 543,75 | 523,75 | 605,25 |
| **Stand desviat** | **551,60** | **135,07** | 135,96 | 131,52 | 150,81 |

Number of photoreceptor survival after white light exposition with and without filter. Unexposed white light group was studied too. The cell count was performed in four retinal eccentricities of each mouse. Fig 4C and the subsequent statistical analysis were done with these data.
